# Inflammatory renal pseudotumor mimicking renal cell carcinoma with complete radiologic resolution: A case report

**DOI:** 10.1016/j.radcr.2026.04.089

**Published:** 2026-06-02

**Authors:** Eusebio Luna Velasquez, Cibele Luna

**Affiliations:** Department of Radiology, Leonard M. Miller School of Medicine, University of Miami, Miami, Florida

**Keywords:** Indeterminate renal mass, Arterionephrosclerosis, Renal cell carcinoma

## Abstract

Small renal masses are increasingly detected incidentally, but up to 25% prove to be benign. Imaging alone often cannot distinguish malignant tumors from inflammatory or ischemic lesions, posing a risk of overtreatment. This case highlights a renal mass initially concerning for malignancy that resolved entirely following treatment for acute pyelonephritis

A 51-year-old woman presented with bilateral flank pain, fever, and myalgias. Laboratory findings were consistent with a urinary tract infection, and cultures grew *Escherichia coli*. Imaging revealed a 4.4 cm right renal mass with features concerning for renal cell carcinoma. However, biopsy demonstrated arterionephrosclerosis without malignancy. Follow-up MRI at 3 months showed complete resolution of the lesion, suggesting a transient inflammatory pseudotumor related to acute pyelonephritis and chronic ischemia. This case illustrates the diagnostic overlap between renal cell carcinoma and benign renal lesions such as focal pyelonephritis, arterionephrosclerosis, or inflammatory pseudotumors. Despite radiologic features mimicking malignancy, clinical context and histology supported a non-neoplastic process. Renal mass biopsy, when combined with follow-up imaging, proved essential to avoid unnecessary intervention. Awareness of inflammatory mimics and their clinical course is critical in managing indeterminate renal lesions, particularly in patients with risk factors for infection or vascular disease. This case underscores the importance of integrating clinical, radiologic, and histologic data in evaluating renal masses. Biopsy and imaging surveillance can help differentiate benign mimics from malignancy, minimizing overtreatment and preserving renal function.

## Graphical abstract

**Figure:**
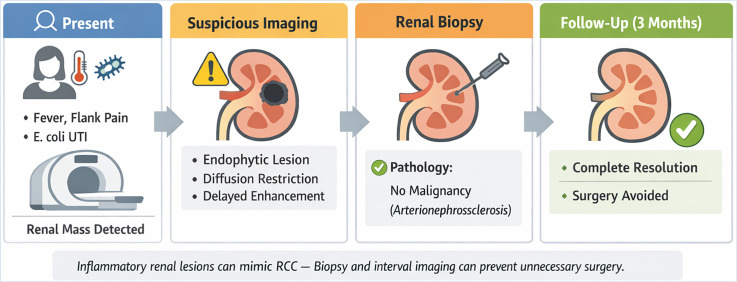

## Introduction

The increasing use of cross-sectional imaging has led to a higher rate of incidental detection of renal masses, with 37% to 61% of renal cell carcinomas (RCC) discovered incidentally [1]. However, approximately 20% to 25% of small renal masses (≤4 cm) are benign on surgical pathology, including oncocytomas, angiomyolipomas, and various inflammatory lesions [1,2].

While contrast-enhanced computerized tomography (CT) and magnetic resonance imaging (MRI) are essential for evaluating renal masses, their ability to distinguish benign from malignant lesions remains limited, as significant overlap exists, particularly with oncocytomas and lipid-poor angiomyolipomas [[2], [3], [4], [5]]. Consequently, renal mass biopsy is increasingly employed for indeterminate lesions where histologic diagnosis may influence management [[6], [7], [8]]. Beyond neoplastic etiologies, various benign conditions, including focal pyelonephritis, xanthogranulomatous pyelonephritis, and IgG4-related disease can mimic malignancy on imaging. [9] Focal pyelonephritis, in particular, may present as a discrete mass with radiographic features resembling RCC [10,11]. Additionally, chronic ischemic changes such as arterionephrosclerosis, often seen in patients with hypertension or renal dysfunction, can result in pseudotumor-like appearances, further complicating accurate diagnosis [12].

Although rare, spontaneous resolution of renal masses has been documented. In observational studies of small renal masses under active surveillance, 5% to 6% of lesions may transiently disappear, likely due to inflammation or artifact, though some later reappear [13]. Importantly, the natural history of these small renal masses is often indolent, with mean growth rates of 0.28 to 0.33 cm per year and metastatic progression in only 1% to 2% of cases [2,13,14].

We present a case of a renal mass initially concerning for RCC based on imaging that resolved entirely following treatment of acute pyelonephritis. This case reinforces the need to consider infectious and inflammatory mimics in the differential diagnosis of renal masses, particularly in patients with signs of systemic infection [3,8,15].

## Case presentation

A 51-year-old woman with a history of hypertension and thyroid cancer (status post thyroidectomy) was admitted with bilateral flank pain, fever, and myalgias. Laboratory testing revealed leukocytosis and a urinalysis consistent with infection; urine cultures grew *Escherichia coli*. She was treated empirically with ceftriaxone 1 gram daily, and culture results confirmed susceptibility to the prescribed antibiotic. However, due to the severity of her symptoms and flank tenderness, a contrast-enhanced CT scan of the abdomen and pelvis was performed (venous phase, 5 mm thickness), revealing a bulky appearance of the right upper pole with surrounding perinephric fat stranding and background cortical scarring, without a clearly defined mass. The area demonstrated hypoenhancement (92 HU) relative to the adjacent renal cortex (115 HU). No discrete striated nephrogram or wedge-shaped appearance was evident (Fig. 1).

**Fig. 1:**
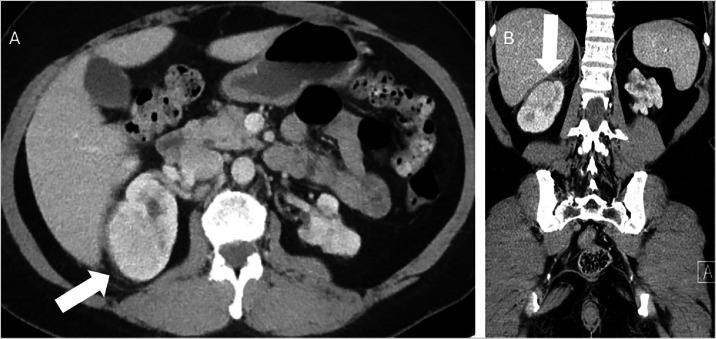
Contrast-enhanced CT demonstrating an indeterminate right upper pole renal abnormality. (A) Axial and (B) coronal venous-phase contrast-enhanced CT images demonstrate a bulky appearance of the right upper pole with surrounding perinephric fat stranding and background cortical scarring (arrows), without a clearly defined mass. The region demonstrates hypoenhancement (92 HU) relative to the adjacent renal cortex (115 HU). Abbreviations: CT, computed tomography; HU, Hounsfield units.

To further characterize the lesion, MRI of the abdomen with contrast was obtained, demonstrating a 3.5 × 4.4 × 4.0 cm right upper pole renal lesion (Fig. 2). The lesion appeared well-circumscribed and predominantly endophytic, T2 isointense, and demonstrated diffusion restriction on DWI (b value: 800)/ADC sequences. On post-contrast imaging, it showed relative hypoenhancement in the arterial phase (50 seconds), became isointense to the surrounding renal parenchyma on the nephrographic/venous phase (100 seconds), and demonstrated a progressive decrease in enhancement on delayed phase imaging (5 minutes). There was no necrosis, hemorrhage, or fat within the lesion. These imaging features raised concern for renal cell carcinoma (RCC), possibly papillary or chromophobe subtype. The left kidney appeared atrophic, with cortical scarring.

**Fig. 2:**
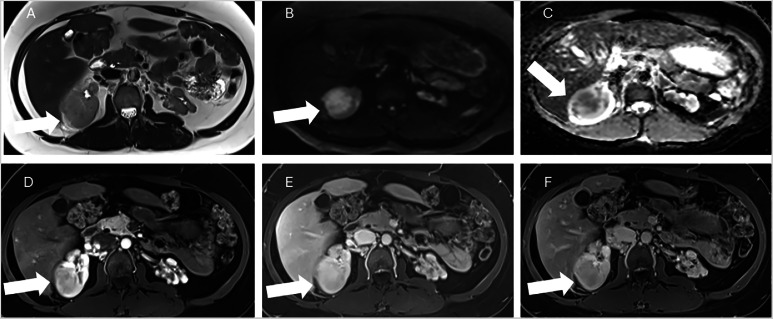
Contrast-enhanced MRI demonstrating a right upper pole renal lesion. Contrast-enhanced MRI of the abdomen. Axial images show a right upper pole renal lesion (arrows). T2-weighted imaging (A) demonstrates an isointense lesion. Diffusion-weighted imaging (b = 800) (B) and corresponding ADC map (C) demonstrate diffusion restriction. On post-contrast T1 fat-suppressed imaging, the lesion demonstrates relative hypoenhancement in the arterial phase (50 seconds) (D), becomes isointense to the background renal parenchyma on the nephrographic/venous phase (100 seconds) (E), and demonstrates a progressive decrease in enhancement on delayed/excretory phase imaging (5 minutes) (F). Abbreviations: ADC, apparent diffusion coefficient; DWI, diffusion-weighted imaging.

Despite radiologic concern for malignancy, the patient’s clinical presentation was atypical for RCC. She denied hematuria, unintentional weight loss, or palpable mass. Given the diagnostic uncertainty and her stable clinical condition, CT-guided core needle biopsy was pursued using an 18G cutting needle coaxially via a 17G introducer needle, targeting the right kidney superior pole. Three core samples were obtained and placed in formalin. Multiple sections of the specimen were prepared, including one H&E, one PAS, one Masson Trichrome, and one Jones’ silver-stained slide. Histopathology revealed arterionephrosclerosis without evidence of malignancy. This unexpected result prompted a decision for close imaging follow-up. Remarkably, repeat MRI at 3 months demonstrated complete resolution of the previously described right upper pole renal lesion (Fig. 3), with no residual mass, abnormal enhancement, or diffusion restriction. This imaging evolution strongly supports a non-neoplastic etiology, most likely representing transient inflammatory changes related to acute pyelonephritis superimposed on chronic ischemic renal injury.

**Fig. 3:**
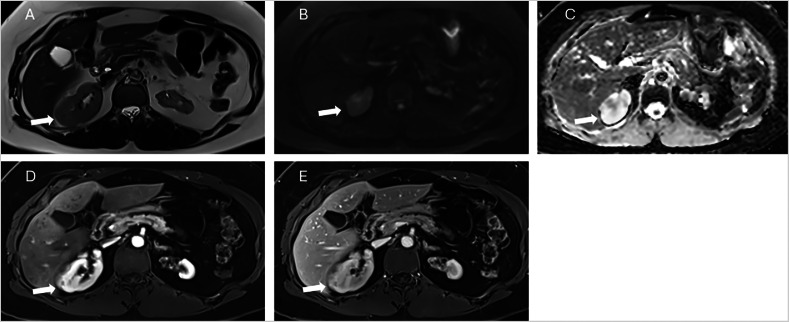
Follow-up contrast-enhanced MRI demonstrating resolution of the previously identified right renal lesion. Follow-up contrast-enhanced MRI of the abdomen obtained 3 months after biopsy. Axial images show T2-weighted imaging (A) with isointense signal in the right renal cortex (arrow). Diffusion-weighted imaging (b = 800) (B) and corresponding ADC map (C) demonstrate no focal areas of restricted diffusion (arrow). Post-contrast T1 fat-suppressed images show isointense enhancement in the arterial phase (50 seconds) (D) and venous phase (100 seconds) (E), without evidence of a residual mass (arrow). Abbreviations: ADC, apparent diffusion coefficient; DWI, diffusion-weighted imaging.

Inflammatory pseudotumors, including focal pyelonephritis related to arterionephrosclerosis, can closely mimic malignant renal neoplasms on imaging. Other important differential considerations include renal infarction, IgG4-related kidney disease, lipid-poor angiomyolipoma, and oncocytoma.

Awareness of these mimics is critical to avoid unnecessary nephrectomy or other interventions that carry substantial morbidity. Tissue diagnosis via biopsy, coupled with interval imaging, can offer a safe and informative approach when imaging findings are indeterminate.

## Discussion

Several benign entities may mimic renal cell carcinoma (RCC) on imaging and subsequently resolve with appropriate management or observation. In the present case, focal pyelonephritis is the most probable diagnosis, given the patient’s acute *E coli* urinary tract infection, febrile illness, and bilateral flank pain, as this condition can present as a discrete mass-like lesion when the inflammatory process is localized [10,11]. Inflammatory pseudotumors (IPT) and IgG4-related kidney disease also warrant consideration, as both can present as renal masses and may demonstrate spontaneous resolution or response to treatment of underlying inflammatory triggers [9,16].

Most IPTs of the kidney are now recognized as a heterogeneous group of entities, and a significant subset of cases previously classified as IPTs are in fact IgG4-related, sharing histopathologic features such as storiform fibrosis, lymphoplasmacytic infiltrate, and obliterative phlebitis [17]. Furthermore, IgG4-related disease is typically accompanied by systemic involvement and characteristic serologic abnormalities, including elevated serum IgG4 levels, hypergammaglobulinemia, eosinophilia, and hypocomplementemia, features that were absent in this case [18].

Other differential entities include renal infarction, which may present with unilateral flank pain and a mass-like appearance; however, it typically demonstrates wedge-shaped perfusion defects rather than a well-defined enhancing mass and is often associated with vascular risk factors or embolic disease [19]. Lipid-poor angiomyolipoma and oncocytoma are benign neoplasms that may mimic RCC on imaging; however, these lesions do not resolve spontaneously and would not be expected to disappear on short-interval follow-up [1,5]. Therefore, the complete radiologic resolution in this case strongly favors a transient inflammatory or ischemic etiology rather than a neoplastic process. The biopsy finding of arterionephrosclerosis further suggests underlying chronic vascular pathology, potentially predisposing to focal ischemia or infarction, conditions that may also produce mass-like lesions with overlapping clinical features [12,20].

Although the lesion exhibited imaging features often associated with RCC, such as solid appearance with diffusion restriction, it lacked additional hallmarks of malignancy and arose in the setting of acute infection [21,22]. The diagnostic overlap between benign and malignant renal lesions, particularly in the case of oncocytomas and lipid-poor angiomyolipomas, limits the specificity of contrast-enhanced CT and MRI [1,5]. Therefore, clinical correlation and percutaneous biopsy are essential when imaging is inconclusive [6].

Renal biopsy in this context proved instrumental in avoiding unnecessary surgery. Modern techniques offer high diagnostic accuracy and low complication rates (0.3%-5.3%). Current guidelines recommend biopsy for indeterminate lesions or in patients with comorbidities or impaired renal function, where histologic confirmation may significantly influence management [6,13]. In this case, biopsy revealed arterionephrosclerosis without malignancy, guiding clinicians toward imaging surveillance. While no active inflammation was noted, this could reflect sampling limitations or the timing of biopsy following antibiotic therapy. Nonetheless, given the clinical presentation and subsequent resolution of the lesion, a conservative approach was deemed appropriate.

The complete resolution observed on 3-month follow-up imaging aligns with the natural history of inflammatory lesions after infection. Approximately 5% to 6% of small renal masses under surveillance may transiently disappear, though around half may later recur, likely reflecting transient inflammatory changes [20,23]. This supports the role of non-operative management in select patients. Active surveillance for small renal masses has shown excellent outcomes, with cancer-specific survival rates nearing 100% and low rates (1%-2%) of metastatic progression during early follow-up [2,6,24].

This case reinforces several clinical principles. First, acute infection should raise suspicion for benign inflammatory processes, even when imaging features suggest RCC [[9], [10]]. Second, renal mass biopsy is critical when the diagnosis may alter management, particularly in patients with comorbidities or solitary functional kidneys [[6], [7]]. Third, follow-up imaging is essential, especially when biopsy results are benign or nondiagnostic [8]. Lastly, awareness of benign mimics of RCC and their clinical behavior can prevent overtreatment, guiding safe and conservative management in patients with indeterminate renal masses [2,9].

## Conclusion

This case demonstrates that renal masses with imaging features suggestive of RCC may represent benign inflammatory or ischemic processes that resolve with treatment of underlying infection or observation. The clinical context of acute pyelonephritis, combined with biopsy findings and complete resolution on follow-up imaging, strongly suggests focal inflammatory changes rather than malignancy. This case reinforces the importance of correlating imaging findings with clinical presentation, utilizing renal mass biopsy for indeterminate lesions, and employing serial imaging to confirm the diagnosis and guide management. Recognition of benign mimics of RCC is critical to prevent unnecessary surgery and preserve renal function, particularly in patients with underlying kidney disease or significant comorbidities.

## Patient consent

Written informed consent was obtained from the patient for publication of this case report and accompanying images. All identifying information has been removed to ensure patient confidentiality.
